# Highlight report: Interspecies extrapolation by physiologically based pharmacokinetic modeling

**DOI:** 10.17179/excli2015-548

**Published:** 2015-09-01

**Authors:** Agata Widera

**Affiliations:** 1 Leibniz Research Centre for Working Environment and Human Factors, IfADo - Ardeystr. 67, D-44139 Dortmund - Germany

## ‭‭⁯

Recently, several articles have been published questioning the usefulness of animal experiments for prediction of human toxicity (Leist and Hartung, 2013[14]). For example, it has been reported that there is almost no correlation of gene expression alterations induced by inflammatory stimuli in humans and mice (Seok et al., 2013[18]). However, a recent study of Thiel et al. (2015[22]) demonstrates that this view may be too pessimistic. Based on pharmacokinetic modeling, Thiel et al. (2015[22]) demonstrated a surprisingly precise mouse to human extrapolation for 10 exemplary pharmaceuticals. For interspecies modeling the authors adjusted four parameter domains in PBPK models: (i) Species-specific physiology, including more than 500 individual parameters, for example organ size and blood flow; (ii) the fraction of non-protein bound test compound; (iii) pharmacokinetic parameters, such as K_m_ and V_max_ for the predominant route of clearance, (iv) tissue specific gene expression of the most important genes responsible for elimination of the test compound (Thiel et al., 2015[22]). Adjusting these parameter domains leads to a very good fit of mouse to human extrapolated plasma concentrations of the test compounds compared to measured human plasma concentrations. One of the limitations of the study of Thiel et al. (2015[22]) is the use of gene expression data for simulation of the influence of metabolizing enzymes and carriers involved in clearance of the test compounds. Since the activities and not RNA levels are relevant in this context, the RNA based approximation can certainly be further improved. However, establishment of a tissue and species specific directory of all relevant metabolizing activities still represents an important future project. 

Currently, much effort is invested in research on *in vitro* systems (Frey et al., 2014[5]; Kim et al., 2015[12]; Hammad and Ahmed, 2014[10]), particularly in the fields of hepatotoxicity (Godoy et al., 2013[8]; Grinberg et al., 2014[9]; Ghallab, 2014[6]; Schug et al., 2013[17]), neurotoxicity (Balmer et al., 2014[1]; Waldmann et al., 2014[23]; Krug et al., 2013[13]; Stöber, 2014[20]) and nephrotoxicity (Giustarini et al., 2009[7]; Faiz et al., 2011[4]). These studies depend on knowledge of *in vivo* relevant concentrations which should be covered by *in vitro* testing. A precise extrapolation of doses *in vivo* to blood concentrations or even better compound concentrations at the target cells of toxicity is therefore critical for progress in the field of alternative methods and can best be achieved by systematic PBPK modeling (Mielke et al., 2011[15]; Sterner et al., 2013[19]; Strikwold et al., 2013[21]; Wang et al., 2000[24]). Further progress may be achieved by combining PBPK models with the recently established spatio-temporal models (Hoehme et al., 2010[11]; Drasdo et al., 2014[2]) which can simulate metabolism at the level of individual cells (Schliess et al., 2014[16]; Drasdo et al., 2014[3]; Widera, 2014[25]). The here discussed study of Thiel and colleagues improves the reliability of extrapolating compound concentrations from mice to human by the systematic adaptation of species specific model parameters and therefore is of high relevance not only for the planning of first-in-man studies but also for an improved use of *in vitro* systems for prediction of human toxicity. 
